# Supplementary material for: An in vivo gene amplification system for high level expression in Saccharomyces cerevisiae
Source: Nat Commun. 2022 May 24;13:2895. doi: 10.1038/s41467-022-30529-8 (PMC9130285; doi:10.1038/s41467-022-30529-8)
Supplement: Supplementary file 1 — Supplementary Information [file 41467_2022_30529_MOESM1_ESM.pdf]

An *in vivo* gene amplification system for high level expression in *Saccharomyces cerevisiae*

Peng *et al*.

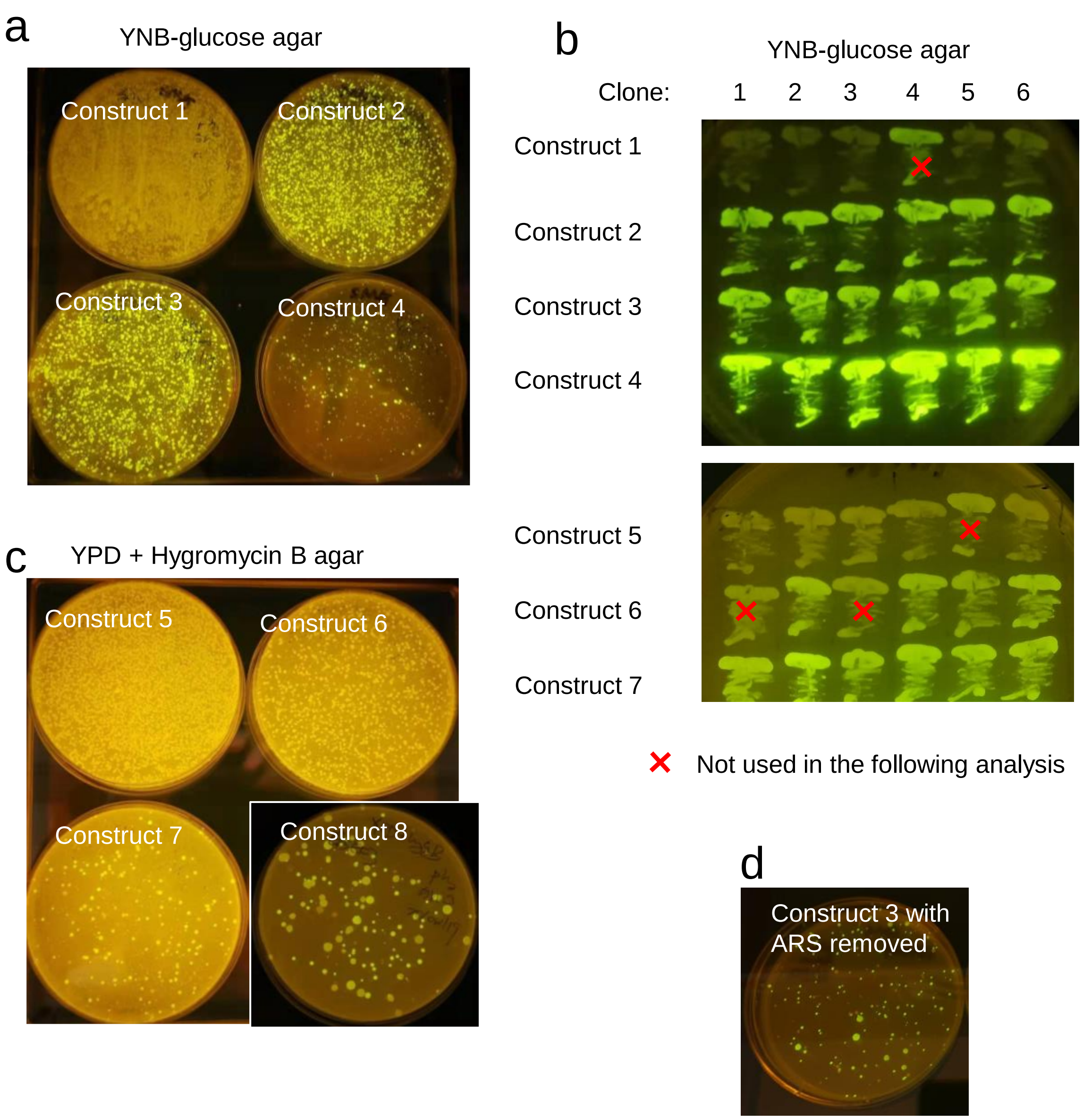

Supplementary Figure 1. Imaging the transformation plates of yeast transformed with the constructs shown in **Figure 2a** using Safe Imager™ 2.0 Blue Light Transilluminator. Photos were taken using a smartphone. The clones in **b** were selected by choosing the random clones for Construct 1 and Construct 5, and bright clones for Construct 2-4 and Construct 6-7 in **a** or **c**. Selection of Construct 8 was not imaged. **d** Fluorescence imaging of transformation plate of Construct 3 with ARS removed.

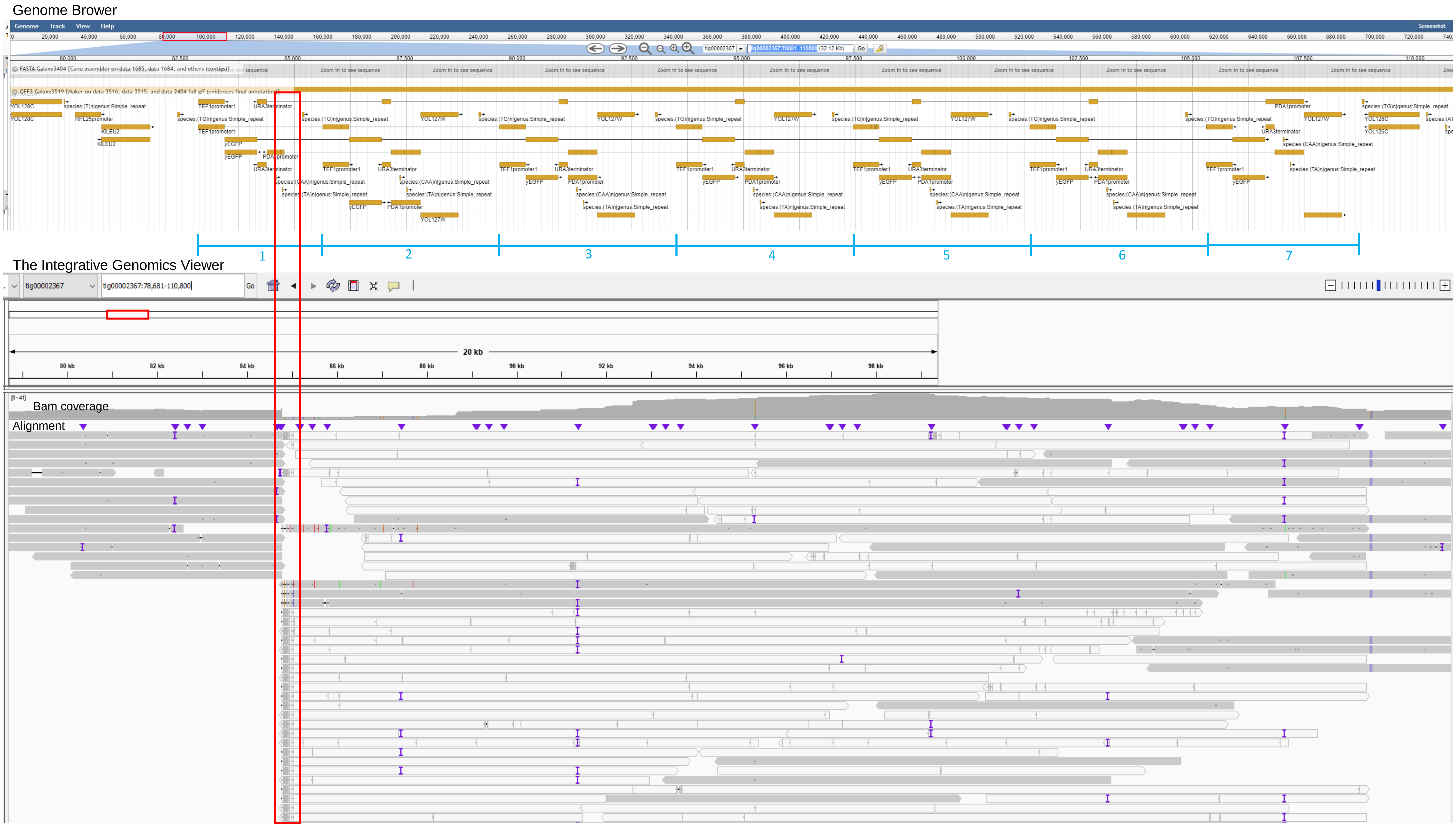

Supplementary Figure 2. Genome structure assembled via Canu assembler at YOL127W (*RPL25*) locus in strain G3AG5 (Construct 3, Figure 2) and alignment with trimmed minION reads outputted by Canu assembler. Red box indicates the sequence might be assembled without sufficient evidence.

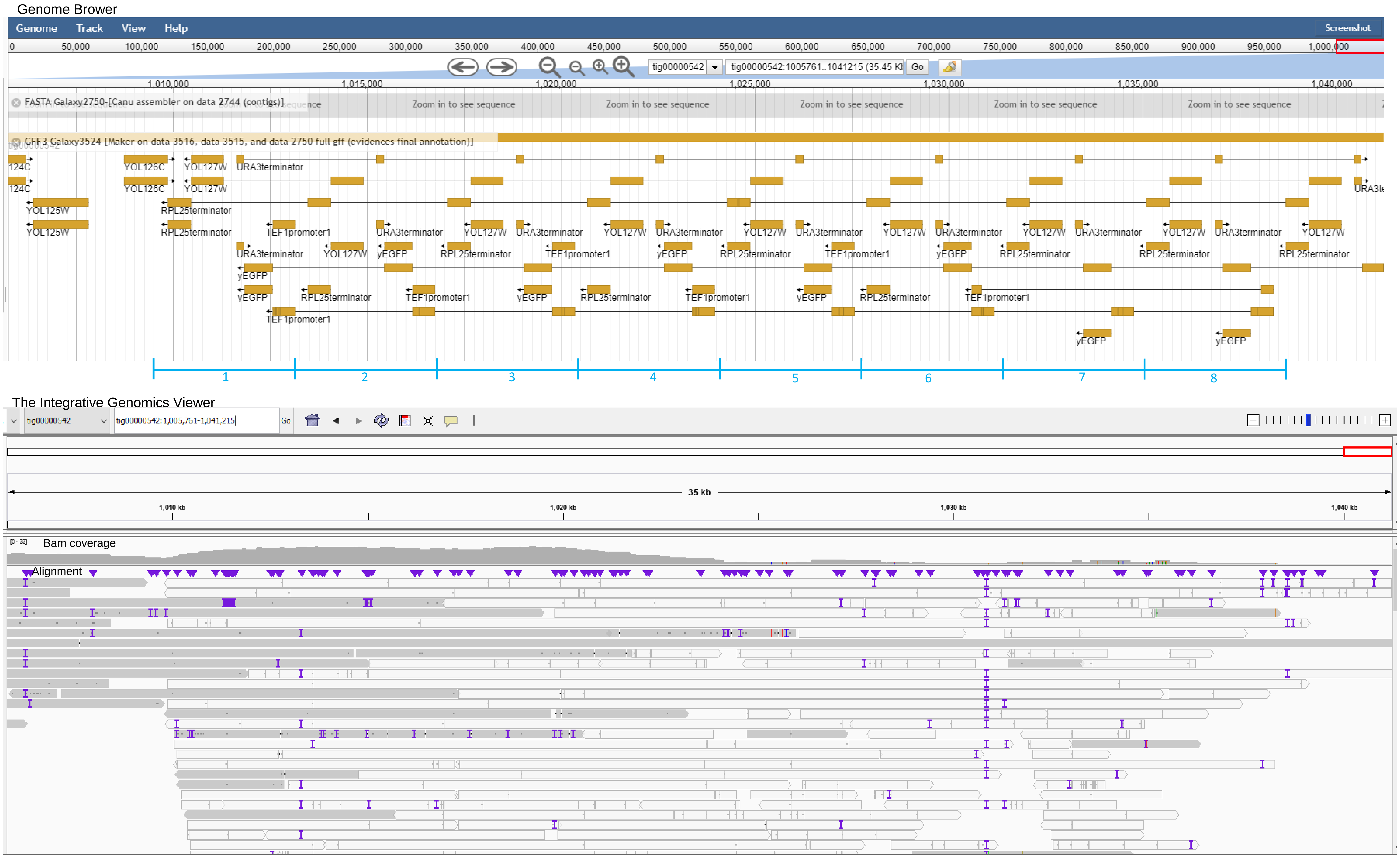

Supplementary Figure 3. Genome structure assembled via Canu assembler at YOL127W (*RPL25*) locus in strain G3AA5 (Construct 4, Figure 2) and alignment with trimmed minION reads outputted by Canu assembler.

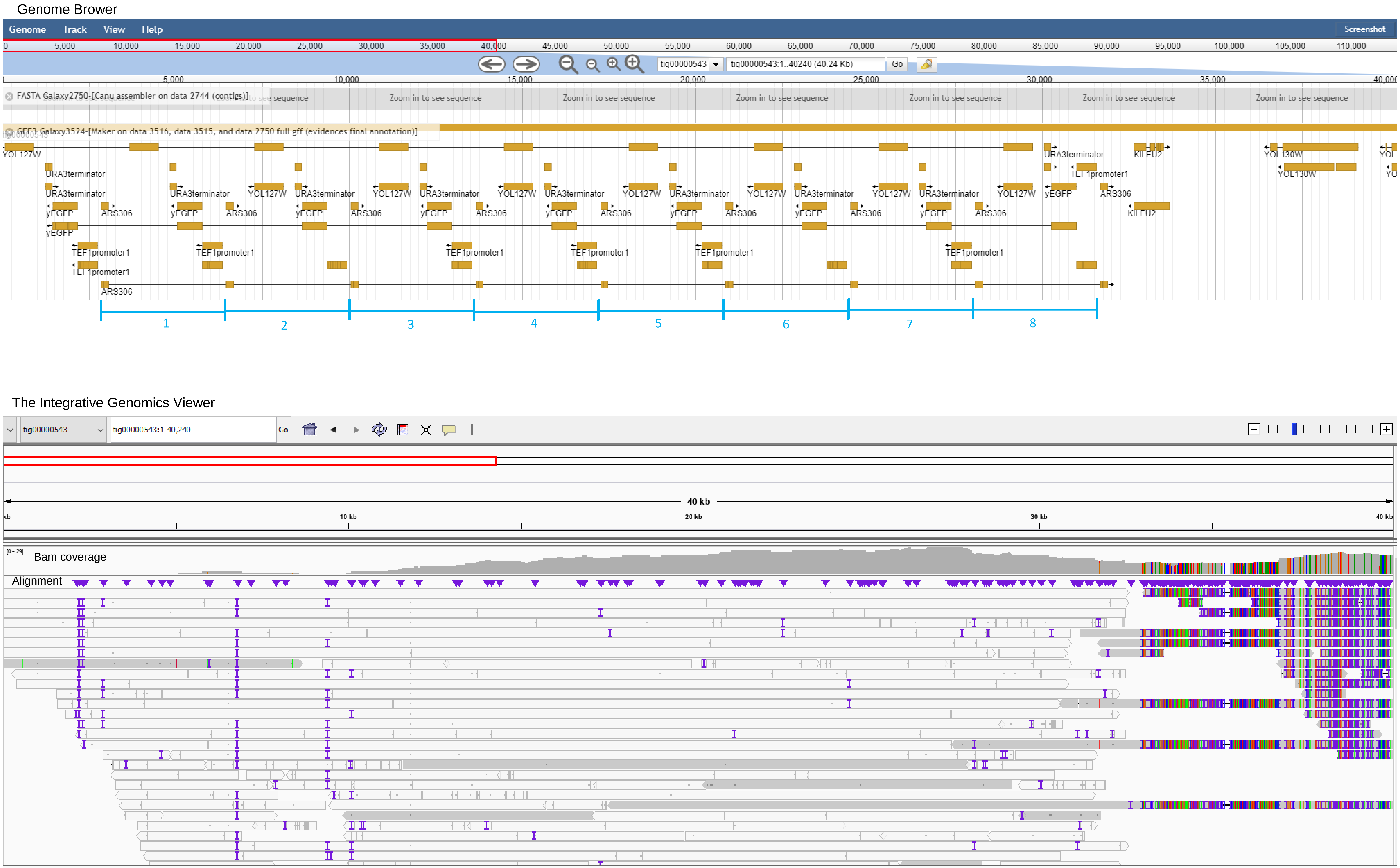

Supplementary Figure 4. Genome structure assembled via Canu assembler at YOL127W (*RPL25*) locus in strain G3AA5 (Construct 4, Figure 2) and alignment with trimmed minION reads outputted by Canu assembler.

Genome Brower

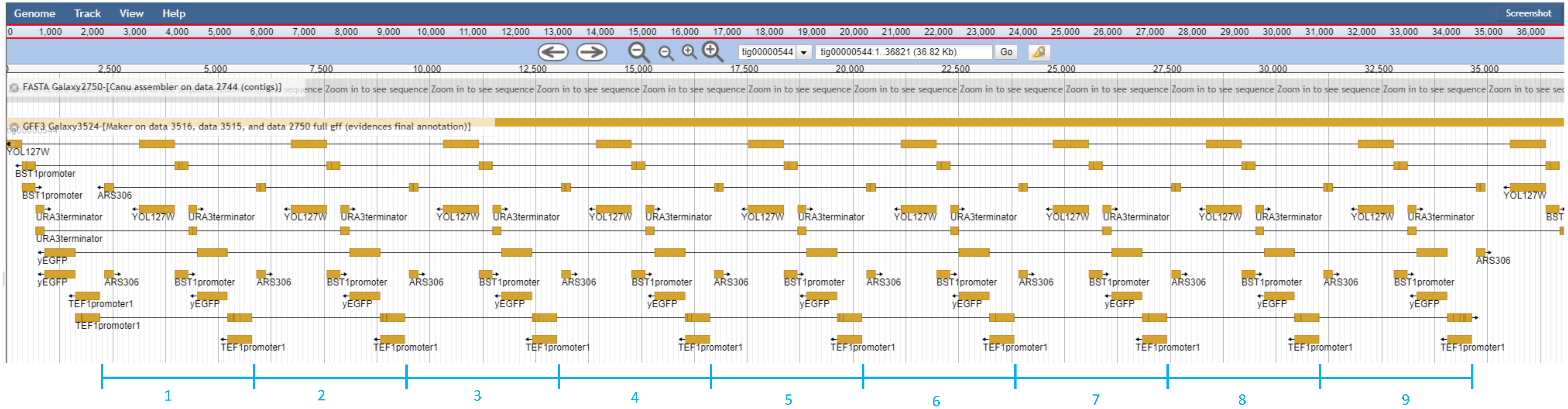

The Integrative Genomics Viewer

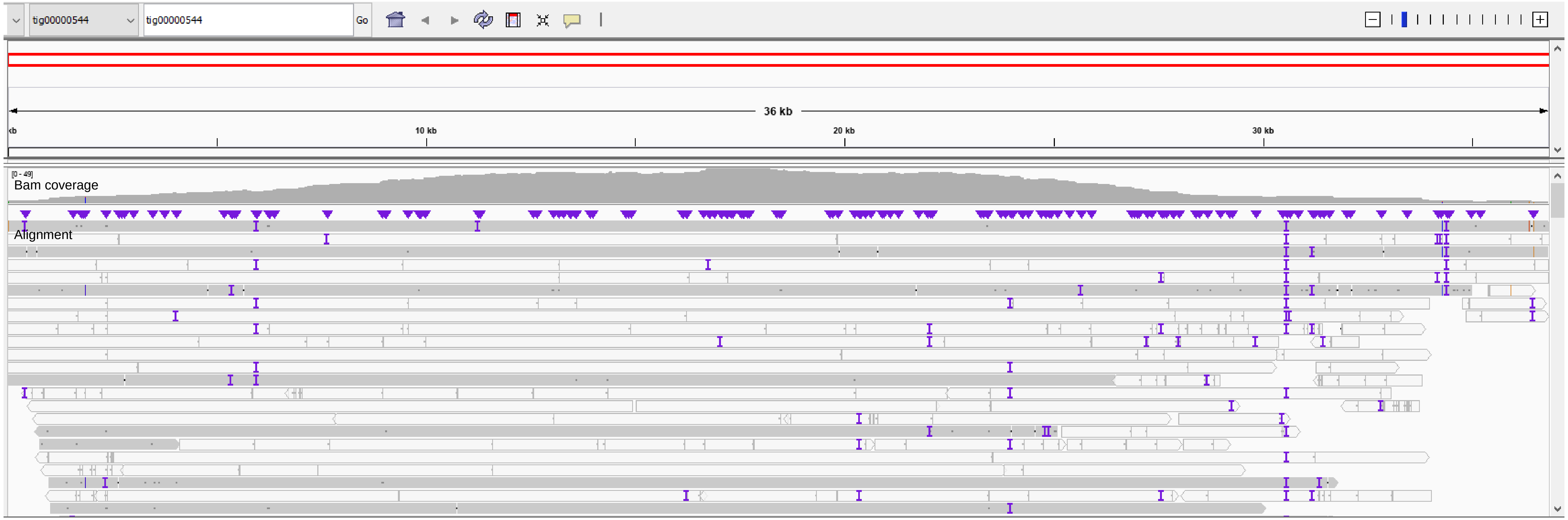

Supplementary Figure 5. Genome structure assembled via Canu assembler at YOL127W (*RPL25*) locus in strain G3AA5 (Construct 4, Figure 2) and alignment with trimmed minION reads outputted by Canu assembler.

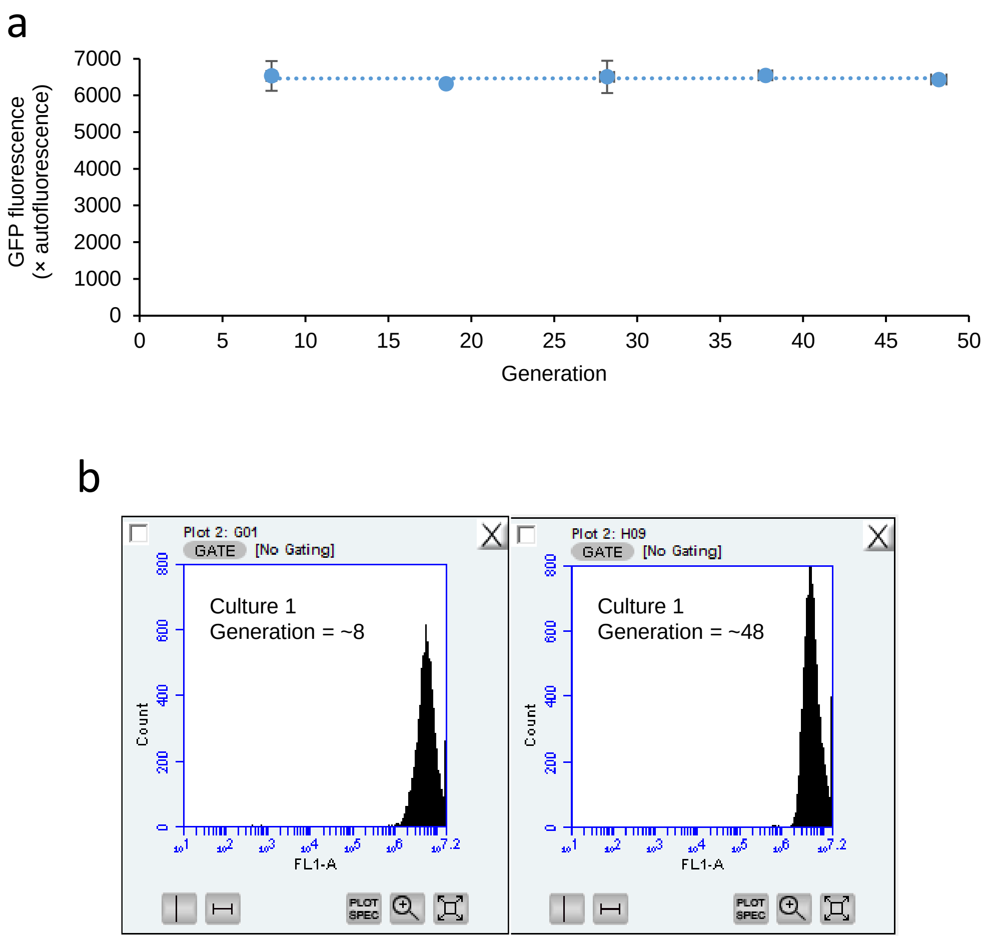

Supplementary Figure 6. Stability test of the strain G3AA5 expressing yEGFP via P_{BTS1}-*RPL25* HapAmp construct (Construct 4, Figure 2). **a** GFP fluorescence levels in the cells sampled from the transferring subcultures. was used in sub-cultivations. For each transferring subculture, cells was inoculated in Yeast extract-Peptone-Glucose (YPD) medium to OD600 equalling to 0.004, grown overnight to OD600 ~ 1 for flow cytometry analysis, and further grown to 24 h to start the next subculture. Mean values ± standard deviations are shown (N = 4 independent cultivation). **b** Flow cytometry profiles of cells at the 8th and 48th generations in subcultures. Source data are provided as a Source Data file.

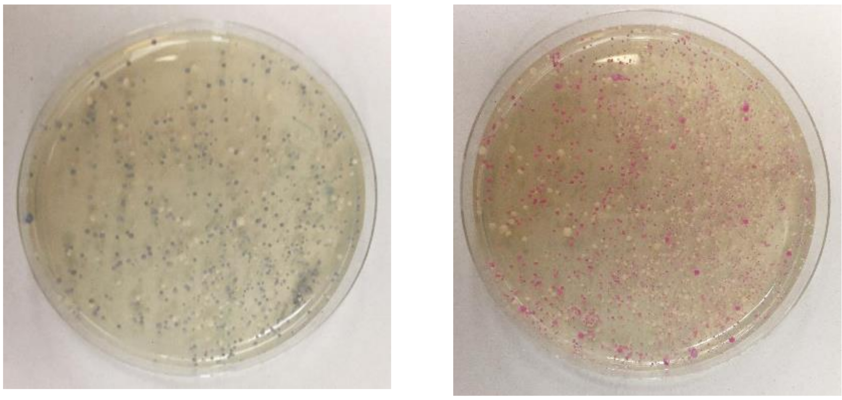

AeBlue EforRed

Supplementary Figure 7. Images of agar plates of yeast transformed with chromoprotein AeBlue or EforRed.

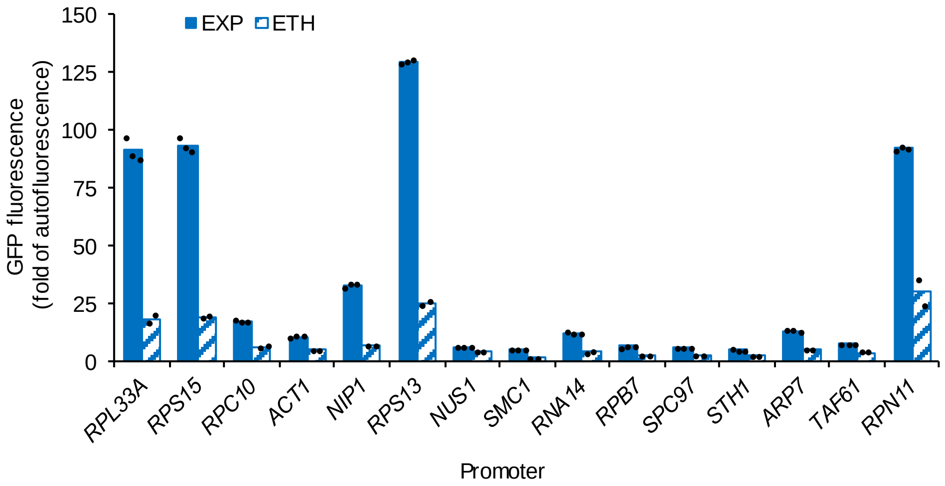

Supplementary Figure 8. Promoter characterisation using yeast enhanced green fluorescent protein (yEGFP) as the reporter in the cells at the exponential growth phase (EXP) and the post-diauxic-shift growth phase (ETH) when ethanol was used as the carbon source. Yeast cells were grown in microplates and yEGFP fluorescence is expressed as percentage of exponential-phase auto-fluorescence of the reference strain. Mean values are shown (N ≥ 2 independent biological replicate). Source data are provided as a Source Data file

Supplementary Method 1. Proteomics analysis

Gel was cutted into 1 x 1 mm cubes and placed into a 0.5 mL tube, followed by adding 100 µL of 100 mM NH4HCO3/acetonitrile. The tubes incubate for 30 minutes with constant shaking. Gel pieces were dehydrated by adding 500 µL neat acetonitrile, vortex to mix, and incubate at room temperature until the gel pieces shrinking into an opaque-white colour.The solution is then removed.

Then the gel pieces were reduced by adding 4 µL aliquot of 500mM of dithiothreitol (DTT) and heating for 60 min at 70 °C. After spin down, 500 µL of neat acetonitrile, incubate for 10 mins and then remove all liquid. Following, the alkylation of cysteines was conducted by addition 8 µL 500mM of iodoacetamide (IAA) was added and tubes were incubated in the dark for 30 min, followed by dding 300 µL of acetonitrile. then all the liquid was removed. Rehydrate gel particles in digestion buffer containing 2 µg trypsin and 25 µL of 50 mM ammonium bicarbonate pH=8.

The reaction is incubated overnight at 37 °C in an oven. 1 µL Fomic acid was added to stop enzyme reaction with incubation for 15 mins at 37 °C in ultrasonic baths, then collected all the digestion liquid to a new tube. This was followed by adding 35 µL of 60% acetonitrile (twice) to each digest tube and sonicating for 60 seconds. After spin down, the liquid supernatant was transferred to the respective new tube. Extracted peptides was dried down in a rotary evaporator and resuspended in 5% acetonitrile (ACN) + 0.1 % formic acid (FA) prior to liquid chromatography.

The peptides were analysed by Liquid chromatography-mass spectrometry (LCMS). The HPLC system was an Ultimate 3000 RSLCnano (ThermoFisher, Germany), and the mobile phase was H2O (A) and 80% ACN (B), both in 0.1% formic acid. An aliquot of 2 µL sample was injected onto a trap column used for online desalting and washed at 20 µL/min prior to a resolving C18 nanoEaseTM M/Z CSH c18 1.7µm (300 µm x 100 mm) column (Waters) using a 60 mins gradient of 8 to 95 % acetonitrile in 0.1 % formic acid at 300 µL/min flow rate.

The eluted peptides were electro-sprayed into a Thermo Orbitrap Q Exactive HF Hybrid Quadrupole-Orbitrap Mass Spectrometer (ThermoFisher, USA). The MS parameters were for MS1 a resolution of 60,000 with scan range at 100-800 m/z. For MS2, the isolation window was 2.0 m/z, the TopN at 40, resolution at 75,000, the nAGC target at 1e6 and the max IT 40 ms.

The post analysis was performed against the yeast database using the ThermoFisher Proteome discoverer software (version 2.4).
