# Supplementary material for: An in vivo gene amplification system for high level expression in Saccharomyces cerevisiae
Source: Nat Commun. 2022 May 24;13:2895. doi: 10.1038/s41467-022-30529-8 (PMC9130285; doi:10.1038/s41467-022-30529-8)
Supplement: Supplementary file 3 — Description of Additional Supplementary Files [file 41467_2022_30529_MOESM3_ESM.pdf]

**Description of Additional Supplementary Files**

File Name: Supplementary  Data 1
Description: Proteomics data for SDS-PAGE (sodium dodecyl sulphatepolyacrylamide gel electrophoresis) band d1 in Figure 6.

File Name: Supplementary Data 2
Description: Proteomics data for SDS-PAGE (sodium dodecyl sulphate- polyacrylamide gel electrophoresis) band d2 in Figure 6.

File Name: Supplementary Data 3
Description: Proteomics data for SDS-PAGE (sodium dodecyl sulphatepolyacrylamide gel electrophoresis) band d3 in Figure 6.

File Name: Supplementary Data 4
Description: Proteomics data for SDS-PAGE (sodium dodecyl sulphatepolyacrylamide gel electrophoresis) band d4 in Figure 6.

File Name: Supplementary  Data 5
Description: Plasmids used in this work.

File Name: Supplementary Data 6
Description: Saccharomyces cerevisiae strains used in this work

File Name: Supplementary Data 7
Description: List of primers and DNA fragments used in this work. PXXX and TXXX indicate promoter and terminator sequence of gene XXX, respectively; sequences in italic and red indicate sequences complimentary to the DNA template.

File Name: Supplementary Data 8
Description: Construction of the plasmids used in this work. Numbers refer to DNA fragments listed in Supplementary Data 7.

File Name: Supplementary Data 9
Description: Construction of the ILHA series strains used in this work. Plasmids refer to Supplementary Data 5. DNA fragments refer to Supplementary Data 7.
